# Boiled or roasted? Bivalve cooking methods of early Puerto Ricans elucidated using clumped isotopes

**DOI:** 10.1126/sciadv.aaw5447

**Published:** 2019-11-27

**Authors:** Philip T. Staudigel, Peter K. Swart, Ali Pourmand, Carmen A. Laguer-Díaz, William J. Pestle

**Affiliations:** 1 School of Earth and Ocean Sciences, Cardiff University, Main Building, Park Place, Cardiff CF10 3AT, UK.; 2 Department of Marine Geosciences, Rosenstiel School for Marine and Atmospheric Science, University of Miami, Miami, FL, USA.; 3 Division of Behavioral and Social Sciences, Valencia College, Orlando, FL, USA.; 4 Department of Anthropology University of Miami, Miami, FL, USA.

## Abstract

Cooking technique reflects a combination of cultural and technological factors; here, we attempt to constrain bivalve cooking temperatures for a pre-Columbian Puerto Rican native population using carbonate clumped isotopes. Analyses of 24 bivalve specimens (*Phacoides pectinatus*) from a shell midden in Cabo Rojo, Puerto Rico, suggest that samples were heated up to 200°C, indicating that roasting rather than boiling may have been the preferred cooking technique. More than half of analyzed samples exhibited a distinct change from modern uncooked shells, possibly reflecting different cooking techniques or the use of a single method wherein shells are unevenly heated, such as when placed on a heated surface. Roasting bivalves would not necessitate the use of ceramic technologies, an observation concurrent with the absence of such artifacts at this site.

## Teaser

Analyses of bivalves suggest peak heating temperatures of 200°C, giving insight into ancient culinary techniques.

## INTRODUCTION

**Fig. 1:**
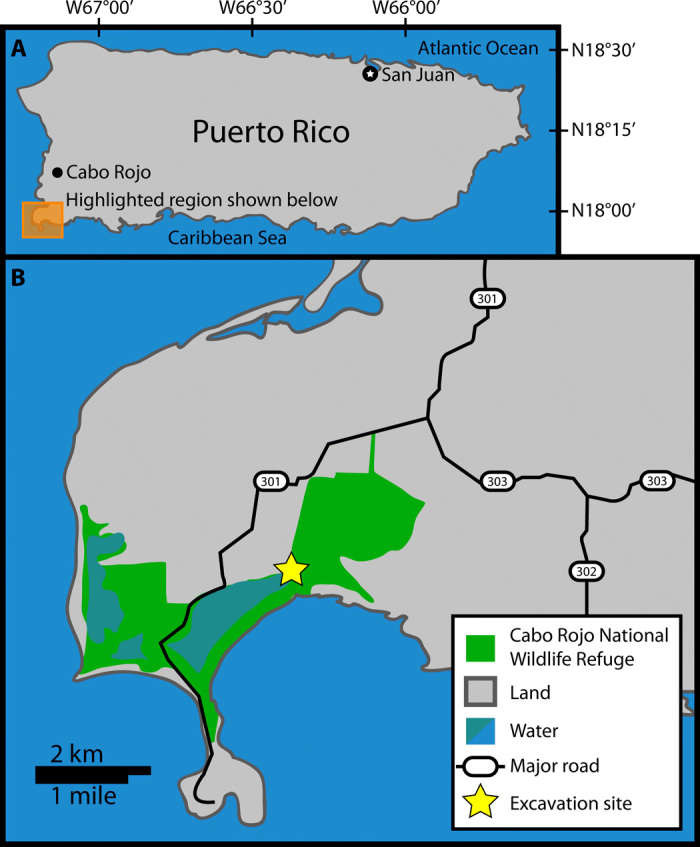
Location figure. (**A**) Map of Puerto Rico with highlighted region. (**B**) Details of the highlighted region showing the Cabo Rojo National Wildlife Refuge and the location of the excavation site discussed here (CRNWR_P13). The figure was adapted from park data published by the U.S. Fish and Wildlife Service.

Investigation of ancient culinary techniques gives valuable insight into the technologies available to ancient cultures. Food preparation techniques can be studied by examination of written accounts (*1*, *2*), excavated food preparation technology (*3*–*5*), and chemical analyses of archaeological sites and food waste (*5*–*8*). Here, we present a study wherein peak cooking temperatures of the bivalve *Phacoides pectinatus*, recovered from a pre-Columbian shell midden in Cabo Rojo, Puerto Rico (Fig. 1), are determined using the distribution of clumped isotope bonds (^13^C─^18^O) in the aragonite shells. The enrichment of ^13^C─^18^O bonds in CO_2_ liberated from carbonate is expressed using the parameter ∆_47_, which is defined as follows

$$\Delta_{47}=[(\frac{R_{\text{measured}}^{47}}{R_{\text{stochastic}}^{47}}-1)-(\frac{R_{\text{measured}}^{46}}{R_{\text{stochastic}}^{46}}-1)-(\frac{R_{\text{measured}}^{45}}{R_{\text{stochastic}}^{45}}-1)]\times 1000‰$$

R^X^ refers to the ratio of mass-X CO_2_ relative to mass-44, as measured using isotope ratio mass spectrometry, and the subscript refers to measured values or predicted ratios in a stochastically distributed gas.

The motivation of this study was to determine whether boiling, which may require the use of ceramic vessels, or roasting, which has no substantial technological requirement, was the preferred cooking method for the early inhabitants of Puerto Rico. Recent studies (*9*, *10*) investigating the abundance of clumped isotope bonds (^18^O─^13^C) in the CO_3_^2−^ ion in biogenic aragonite (CaCO_3_) reveal that the moderate heating (125° to 250°C) used in common culinary preparation techniques is sufficient to change the abundance of these bonds. The extent of this alteration is proportional to the duration of heating and peak heating temperature, and thus the clumped isotope distribution in the material (∆_47_) can be measured and used to infer the temperature of cooking, giving insight into the technique used. Whereas this alteration process is fairly rapid at temperatures in excess of 175°C and thus the duration of heating can be ignored, cooler temperatures result in some ambiguity in constraining heating durations (*10*). Experiments wherein samples are heated to 100°C result in no significant change because of this kinetic limitation. Consequently, boiling samples is not expected to measurably affect the isotopic composition of aragonite, although dissolution of aragonite and precipitation of secondary calcite may still occur and affect the final composition (*11*, *12*).

**Fig. 2:**
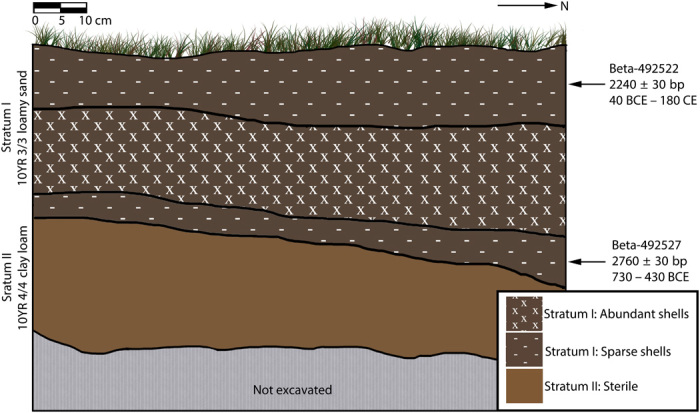
Stratigraphic section of shell midden excavation site including location of radiocarbon age estimates.

The carbonate clumped isotope technique is applied here to study the cooking temperatures for bivalves in a prehistoric shell midden site in Puerto Rico (Fig. 2) as a proxy for cooking method. The patterning of material culture at the site, combined with radiocarbon determinations, associates the site with the Archaic/pre-Arawak inhabitation of the island, which lasted from ca. 4300 BCE to 200 CE. Many of these sites are located near present-day coastlines and are typified by high concentrations of marine gastropods and bivalves, as well as abundant lithics and low abundance of ceramics, when present (*13*–*20*). While the presence and/or ubiquity of ceramic technologies in the cultural tool kit of the Archaic Puerto Rican people remains an open question, the food preparation techniques of later Arawak people commonly included boiling or preparation of soups in ceramic vessels (*19*–*22*). The clumped isotope method offers a previously unexplored approach for determining whether such methods (e.g., boiling in ceramic containers) were commonplace among Archaic people as well. While it is possible to boil water without ceramic vessels (e.g., by placing heated stones in earthen pits or pitch-lined baskets), a negative finding (i.e., that roasting, rather than boiling, was the preferred cooking technique) would offer circumstantial evidence against the ubiquity of ceramic technology among the pre-Arawak Puerto Rican people. Bivalves, which account for 87% of preserved mass of the animal remains recovered from the site, are typically prepared for human consumption by boiling or dry roasting. Here, we assess whether boiling (possibly in ceramic vessels) or roasting was the preferred means of cooking bivalves as a proxy measure for the possession of ceramics by the population who produced the shell midden.

### RESULTS

All shell midden constituents were analyzed using x-ray diffractometry (XRD) and confirmed to be >95% aragonite (*23*); the nominal precision of this method is ±5%. The implementation of a separate calibration scheme (discussed in the Supplementary Materials) results in a change in calcite content by less than 1% from the aforementioned values. Conversion from aragonite to calcite would imply either post-depositional dissolution of aragonite and subsequent precipitation of calcite or heating in excess of 350°C, which would facilitate the solid-state conversion from one mineral to another accompanied by a significant decrease in the materials’ ∆_47_ values (*10*). At 350°C, ~10% of aragonite will convert to calcite over approximately 30 min; at 400°C, this same degree of alteration would occur over approximately 5 min (*10*). The absence of conversion from aragonite to calcite has historically been inferred to represent that these alteration processes never occurred (*11*) and thus any heating was below the threshold for the mineral conversion.

The isotopic compositions of all shell samples are presented in Table 1. The carbon isotope ratio (δ^13^C) of shell midden constituents did not vary significantly between ancient samples, although modern shells were more variable in composition, a result likely attributable to changes in land utilization in the region, which could affect relative supply of marine carbon and organic carbon. The oxygen isotope ratios (δ^18^O) were not statistically significantly different given the number of analyses (*P* > 0.05). The average δ^18^O value in all samples was −0.1‰ relative to the Vienna Pee Dee belemnite (PDB) standard and ranged between 0.7 and −0.7‰, respectively.

**Table 1:** Average δ^13^C, δ^18^O, and Δ_47_ values for all 30 sample materials. Uncertainty corresponds to ±1 SD from replicate analyses. Inferred paleotemperatures were calculated from Δ_47_ values using the calibration from Staudigel *et al.* (*25*). ARF, absolute reference frame; VPDB, Vienna PDB.

			**δ^13^C**		**δ^18^O**		**Δ_47_**		**Inferred** **paleotemperature**
**Sample group**	**ID**	***N***	**(‰VPDB)**	**±**	**(‰VPDB)**	**±**	**(‰ARF)**	**±**	**(°C)**
CR_Modern	A	2	3.558	0.052	−0.126	0.092	0.673	0.004	32.7
CR_Modern	B	1	−2.775		0.166		0.692		26.1
CR_Modern	C	1	−2.911		0.280		0.675		32.0
CR_Modern	D	2	−3.196	0.025	0.307	0.130	0.687	0.000	27.8
CR_Modern	E	1	4.708		−0.448		0.701		23.2
CR_Modern	F	1	1.730		0.710		0.694		25.4
**CRNWR_P13 (shell midden) samples**									
Level 1.1	A	1	3.413		−0.097		0.687		27.8
Level 1.1	B	1	2.954		−0.431		0.687		27.8
Level 1.1	C	1	2.866		0.195		0.681		29.8
Level 1.1	D	2	4.244	0.033	−0.570	0.012	0.598	0.047	63.3
Level 1.1	E	2	4.412	0.021	−0.429	0.044	0.597	0.008	64.1
Level 1.1	F	2	4.241	0.069	−0.077	0.031	0.639	0.010	45.3
Level 1.2	A	2	3.123	0.079	0.062	0.067	0.704	0.019	22.1
Level 1.2	B	1	3.679		0.061		0.660		37.2
Level 1.2	C	2	3.776	0.005	−0.748	0.049	0.507	0.044	117.6
Level 1.2	D	1	4.029		−0.395		0.679		30.5
Level 1.2	E	2	3.494	0.007	0.084	0.083	0.639	0.001	45.6
Level 1.2	F	2	3.234	0.152	0.113	0.075	0.573	0.027	76.0
Level 1.3	A	2	2.378	0.091	0.168	0.143	0.620	0.019	53.5
Level 1.3	B	2	1.872	0.011	−0.236	0.006	0.561	0.022	82.4
Level 1.3	C	2	3.456	0.071	0.030	0.028	0.689	0.007	27.1
Level 1.3	D	2	4.020	0.039	−0.212	0.008	0.689	0.006	27.1
Level 1.3	E	1	3.366		0.147		0.688		27.4
Level 1.3	F	1	3.039		0.033		0.685		28.2
Level 1.4	A	2	3.273	0.022	−0.028	0.085	0.706	0.026	21.3
Level 1.4	B	2	1.645	0.040	−0.482	0.028	0.634	0.005	47.7
Level 1.4	C	2	3.290	0.002	−0.028	0.014	0.544	0.021	92.7
Level 1.4	D	2	4.260	0.034	−0.516	0.001	0.586	0.004	69.3
Level 1.4	E	2	4.084	0.013	−0.495	0.048	0.657	0.003	38.4
Level 1.4	F	1	3.974		0.178		0.672		32.9

**Fig. 3:**
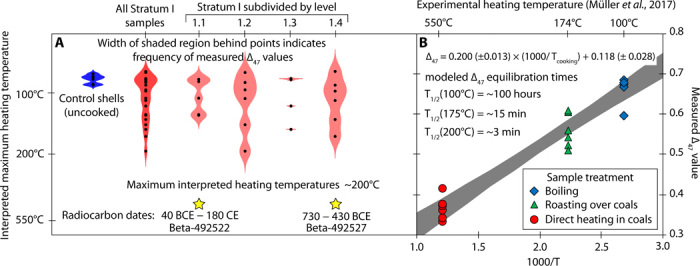
Δ_47_ values for uncooked modern shells and shell midden constituents from CRNWR and experimental results for cooked bivalves presented by Müller *et al*. (*9*). (**A**) Δ_47_ values of all modern and ancient samples measured in this study (black dots); colored regions display the probability distribution for each group of analyses. Shaded regions behind points delineate sample frequency and are generated using distributionPlot for MATLAB by J. Dorn. The location, estimated age ±2σ, and sample ID of radiocarbon measurements are shown in the bottom of the figure as stars. (**B**) Δ_47_ values (*y* axis) and temperature of heating (*x* axis) of Müller *et al.* (*9*) cooked clams. T_1/2_ estimates for Δ_47_ equilibration half-lives based on the Arrhenius model extrapolating through direct heating experiments of biogenic aragonite between 125° and 175°C (*10*). The secondary *y* axis for both figures is estimated maximum cooking temperature.

Modern samples’ ∆_47_ values ranged between 0.70 and 0.67‰ in the thermodynamically defined absolute reference frame (*24*). On the same scale, bivalves collected from the shell midden ranged between 0.71 and 0.51‰, with 10 falling within the range of modern samples and 14 exceeding this range toward more negative values (Fig. 3A). ∆_47_ values for shells in Stratum I were significantly more variable than modern specimens, and *F* values (σ_midden_^2^/σ_modern_^2^) for shell midden samples compared to modern samples range between 15 and 44 (*n* = 6 for each population, *P* = 0.005 to 0.0003). Levels 1.1 through 1.4 all had statistically similar variance to one another. A combined *t* test between all samples recovered from Stratum I (*n* = 24) and the control group shells (*n* = 6) indicates a highly significant (*P* < 0.001) difference in mean ∆_47_ value between the two populations. There is no significant difference between the left and right valves’ ∆_47_ values among shell midden constituents.

## DISCUSSION

The ∆_47_ values of the modern control group bivalves fall within a narrow range between +0.70 and +0.67‰, which corresponds to a temperature range of 23° to 32°C when using the temperature-∆_47_ calibration generated using the same instrumentation (*25*). This temperature range is consistent with seasonal variation in the region (*26*). In contrast, the ∆_47_ values in the shells from the shell midden corresponded to paleotemperatures between 21° and 118°C. Evidently, the warmer temperature estimates significantly exceed the natural temperature range, indicating that the ∆_47_ values may have been reequilibrated during a heating period, which we interpret as having occurred during cooking before consumption and deposition. Whereas wildfires are a distinct possibility at this site, the compact and relatively unmixed stratigraphy of the deposit and lack of highly thermally altered shells in the study sample argues against the occurrence of high-temperature (up to 800°C) grass fires; thus, post-burial heating was likely minimal and unlikely to have affected the ∆_47_ values of shell midden constituents. Of the 24 shells analyzed, 14 have ∆_47_ values less than the average ± 1 SD for modern-day shells (0.689 ± 0.011‰), while 10 are within this range or are more positive (Fig. 3A).

The nonuniform distribution of ∆_47_ values between shell midden constituents suggests a nonuniform thermal history for shell midden constituents. This could be a result of one of several scenarios including (i) the discarding of uncooked shells, (ii) the use of different cooking techniques resulting in different thermal histories for different shells, or (iii) a single cooking technique wherein shells are unevenly heated. At temperatures below 150°C, the rate of ∆_47_ resetting in biogenic aragonite necessitates that the duration of heating far exceeds a hypothetical 10-min cooking time (*10*). Cooking methods involving boiling are expected to require hundreds of hours to significantly affect ∆_47_ values (*10*) and, thus, would be indistinguishable from the control group shells. The uneven distribution of ∆_47_ values between valves, where approximately half of the shells remain unchanged, could be produced using a single cooking technique, which causes uneven heating of valves during cooking. If clams are heated from below, heat conducts through the shell, and the bottom valve would be heated significantly more than the top. This grilling technique does not require ceramic technology to prepare the clams for eating and, thus, is consistent with archaeological findings at this site.

The evidence provided from clumped isotopes indicates that approximately 50% of *P. pectinatus* valves at the Cabo Rojo site in Puerto Rico were heated to temperatures well above that of boiling water, with no significant variance with respect to burial depth. The precise reason for this nonuniformity in ∆_47_ values, however, cannot be determined with the available data. Our findings could indicate either two separate preparation processes (or the preparation of only half the valves) or a single process that resulted in uneven heating within the specimen. Nevertheless, it can be clearly demonstrated that most of the shells were heated to temperatures greater than 100°C but no greater than 200°C. Future work implementing the clumped isotope technique in the study of ancient culinary practices would benefit from additional experimentation using different species and culinary preparation techniques.

## MATERIALS AND METHODS

CRNWR_P13 is a 2500-m^2^ shell midden site located within the boundaries of the Cabo Rojo National Wildlife Refuge in southwestern Puerto Rico (Fig. 1). As seen in Fig. 2, excavation of a 1 m × 1 m test unit within the site revealed two distinct soil strata (based on color/texture). Stratum I was an artifact-rich midden deposit, composed of 10YR 3/3 loamy sand, which extended from the present soil surface to a depth of 35 to 45 centimeters below surface (cmbs). Artifacts were present from the outset (denoted by the “-” symbol in Fig. 2), but reached peak density at 15 to 30 cmbs (denoted by “X” in Fig. 2), and decreased thereafter to the end of Stratum I. For purposes of stratigraphic control, Stratum I was excavated as four vertically successive 10-cm levels. Two radiocarbon dates [Beta-492522 (2240 ± 30 bp) and Beta-492527 (2760 ± 30 bp)] were obtained from Stratum I, at the top and bottom of the shell midden deposit, respectively. Calibration of these dates using Calib v7.1 (http://calib.org, accessed 20 March 2019), the Marine13 Curve (*27*), and a local deltaR of −27 ± 24 yields ranges (±2σ) of 30 BCE to 180 CE for Beta-492522 and 730 to 430 BCE for Beta-492527. Stratum II consisted of a 10YR 4/4 clay loam and was almost entirely sterile (with some isolated artifacts having been shifted into this stratum as a result of land-crab burrowing). While bioturbation undoubtedly had occurred, only a minute number of artifacts appear to have been vertically displaced (with only 41 g of shell in Stratum II, as compared with nearly 20 kg in Stratum I). This fact, combined with the overall compactness and robusticity of the midden, as well as the as-expected vertical succession of radiocarbon dates, gives an overall impression of a robust and largely intact midden profile. Products of excavation include an abundance of marine shell (primarily bivalves) and smaller quantities of faunal bone and lithic artifacts. To date, no ceramic artifacts have been encountered at this site.

In total, nearly 20 kg of shell was recovered from the test unit. Shells used in the present analysis were removed from the four 10-cm levels of Stratum I (referred in text and figures as level 1.1 through level 1.4). In total, six randomly selected shells were analyzed from each of these four levels and six additional shells were collected at the modern coastline (50 to 100 m distant from the midden). Selection of shells from successive levels was carried out not as a means of testing changes in cooking technique over time but in an attempt to improve the representativeness of the sample. While the number of samples is small relative to the size of the assemblage, the stratified random sampling approach used was judged as the most effective means of providing for a representative but still manageable number of samples. Bivalves were ground using a ceramic ball-mill grinder into a fine powder and stored in glass vessels. Approximately 50 mg of this powder was made into a water slurry and mounted to 1-cm glass slides for XRD analysis. The calculation of calcite content using the XRD results is discussed further in the Supplementary Materials.

Isotopic analysis was conducted following the methods described in previous manuscripts (*9*, *10*, *25*). Ten to twelve milligrams of carbonate powder were weighted into copper boats and placed in a manually actuated sample carousel on a vacuum line. Samples were dropped into the acid bath and reacted for 30 min; the liberated CO_2_ and water vapor was collected in a trap cooled using liquid nitrogen. Water-CO_2_ separation was facilitated by warming the trap to −90°C with a methanol slush. The CO_2_ was collected in a separate trap, and the residual water was then heated and pumped away. Following convention, the CO_2_ was passed through a column packed with Porapak Q beads and cooled between −20° and −30°C; this removes organic contaminants, which may produce isobaric interference in subsequent analysis. The CO_2_ was frozen in a trap on the other side of the Porapak column, and no carrier gas was used during the gas preparation process. The clean, dry CO_2_ was transferred to a resealable glass vessel and transferred to a Thermo Scientific 253 mass spectrometer for analysis.

Mass spectrometric analysis consisted of six blocks of eight analyses of sample gas bracketed by analyses of the working reference gas, and these blocks were bracketed by similar analyses off peak, to correct for the negative pressure baseline (*28*). “Raw” ∆_47_ values relative to in-house working gas were calculated following the methods outlined by Huntington *et al.* (*29*). CO_2_ gas standards equilibrated with water at 25° and 50°C and CO_2_ equilibrated in a quartz vessel at 1000°C were used as reference gas to correct data into the absolute reference frame (*24*). Stochastic distributions were calculated using the “Brand” parameters for PDB as recommended by Daëron *et al.* (*30*). Replicate analyses of carbonate reference materials used for interlaboratory comparison (ETH 1, 2, 3, and 4) (*31*) yielded ∆_47_ values of 0.288 ± 0.009, 0.296 ± 0.010, 0.683 ± 0.014, and 0.538 ± 0.009, respectively, in the absolute reference frame. Analyses by Schauer *et al.* (*32*) using acid digestion at 90°C yielded ∆_47_ values 0.287, 0.280, 0.694, and 0.553‰, respectively, all of which fall within the uncertainty of our measurements of the same materials. Individual bivalve and ETH standard analyses are presented in data S1. The isotopic data for all bivalves, including paleotemperature estimates, are summarized in Table 1.

The calibration for estimating peak heating temperatures was constructed using data from a previously published study (*9*), which showed a relationship between cooking temperatures and the ∆_47_ value of bivalve shells. These data largely agree with the dataset from a direct heating experiment of sclerosponge aragonite (*10*), suggesting that this effect is common in marine biogenic aragonite.

## Supplementary Material

http://advances.sciencemag.org/cgi/content/full/5/11/eaaw5447/DC1

Download PDF

Data S1

Boiled or roasted? Bivalve cooking methods of early Puerto Ricans elucidated using clumped isotopes

## SUPPLEMENTARY MATERIALS

Supplementary material for this article is available at http://advances.sciencemag.org/cgi/content/full/5/11/eaaw5447/DC1 Supplementary discussion on quantifying mineralogy using XRD Table S1. XRD peak areas for calcite [104] (A_calc_) and aragonite [111] (A_arag_) of calibration materials and bivalves from the CRNWR excavation site. Fig. S1. XRD scans of pure aragonite and calcite. Fig. S2. Relative peak area for aragonite and calcite relative to the molar fraction of each mineral. Fig. S3. Photographs of all shells analyzed in this study. Fig. S4. Cross plot of δ^13^C and δ^18^O values for all modern and shell midden bivalves discussed in the main text. Data S1. Isotopic analyses of equilibrated gases, Cabo Rojo bivalves, and ETH carbonate standards.
