# Supplementary material for: Boiled or roasted? Bivalve cooking methods of early Puerto Ricans elucidated using clumped isotopes
Source: Sci Adv. 2019 Nov 27;5(11):eaaw5447. doi: 10.1126/sciadv.aaw5447 (PMC6957291; doi:10.1126/sciadv.aaw5447)
Supplement: http://advances.sciencemag.org/cgi/content/full/5/11/eaaw5447/DC1 [file supp_5_11_eaaw5447__index.html]

Science Advances | Science AdvancesAAASSearchScience AdvancesMenu

## Supplementary Materials

**The PDFset includes:**

- Supplementary discussion on quantifying mineralogy using XRD
- Table S1. XRD peak areas for calcite 104 (Acalc) and aragonite 111 (Aarag) of calibration materials and bivalves from the CRNWR excavation site.
- Fig. S1. XRD scans of pure aragonite and calcite.
- Fig. S2. Relative peak area for aragonite and calcite relative to the molar fraction of each mineral.
- Fig. S3. Photographs of all shells analyzed in this study.
- Fig. S4. Cross plot of δ13C and δ18O values for all modern and shell midden bivalves discussed in the main text.
- Legend for data S1

Download PDF

**Other Supplementary Material for this manuscript includes the following:**

- Data S1 (Microsoft Excel format). Isotopic analyses of equilibrated gases, Cabo Rojo bivalves, and ETH carbonate standards.

**Files in this Data Supplement:**

- Adobe PDF - aaw5447\_SM.pdf
