# Supplementary material for: Boiled or roasted? Bivalve cooking methods of early Puerto Ricans elucidated using clumped isotopes
Source: Sci Adv. 2019 Nov 27;5(11):eaaw5447. doi: 10.1126/sciadv.aaw5447 (PMC6957291; doi:10.1126/sciadv.aaw5447)
Supplement: Download PDF [file aaw5447_SM.pdf]

Science Advances
AAAS

advances.sciencemag.org/cgi/content/full/5/11/eaaw5447/DC1

Supplementary Materials for

Boiled or roasted? Bivalve cooking methods of early Puerto Ricans elucidated using clumped isotopes

Philip T. Staudigel*, Peter K. Swart, Ali Pourmand, Carmen A. Laguer-Díaz, William J. Pestle

*Corresponding author. Email: staudigelp@cardiff.ac.uk

Published 27 November 2019, *Sci. Adv.* **5**, eaaw5447 (2019)
DOI: 10.1126/sciadv.aaw5447

The PDF file includes:

Supplementary discussion on quantifying mineralogy using XRD
Table S1. XRD peak areas for calcite [104] (A_{calc}) and aragonite [111] (A_{arag}) of calibration materials and bivalves from the CRNWR excavation site.
Fig. S1. XRD scans of pure aragonite and calcite.
Fig. S2. Relative peak area for aragonite and calcite relative to the molar fraction of each mineral.
Fig. S3. Photographs of all shells analyzed in this study.
Fig. S4. Cross plot of $\delta^{13}C$ and $\delta^{18}O$ values for all modern and shell midden bivalves discussed in the main text.
Legend for data S1

Other Supplementary Material for this manuscript includes the following:

(available at advances.sciencemag.org/cgi/content/full/5/11/eaaw5447/DC1)

Data S1 (Microsoft Excel format). Isotopic analyses of equilibrated gases, Cabo Rojo bivalves, and ETH carbonate standards.

Supplementary discussion on quantifying mineralogy using XRD

The methods and calibration used for determining aragonite/calcite in this study are taken from the inter-laboratory study presented by Swart et al., (2003). The results of this method were verified for this study by measuring mixtures of aragonite and calcite with known proportions. The aragonite reference material was a ~1 cm^3 piece of sclerosponge (*Ceratoporella nicholsoni*) which was crushed using a mortar and pestle, this is the same material used in a previous study (Staudigel and Swart 2016). The calcite reference material was produced by crushing a fragment of a stalagmite from Abaco Island in the Bahamas. These powdered samples were weighed to form molar mixtures of 100%, 75%, 50%, 25% and 0% aragonite. Mixtures deviated slightly from these nominal values, as shown in table S1 and figure S1. Samples were mixed into a slurry with deionized water with a mortar and pestle using identical preparation methods to samples discussed in main text. The slurry was divided between three slides which were analyzed separately.

Samples were measured using a Panalytical™ X-ray diffractometer between 2θ = 23°– 35° with X-rays Cu K-α emission (λ = 1.54Å). The relative areas of the aragonite [111] peak (2θ = 26.1) and the calcite [104] peak (2θ = 29.4) were used to determine the relative fraction of each mineral (Figure S1). The area under each peak ($A_{calcite}$ and $A_{aragonite}$) is proportional to the abundance of the relevant mineral ($F_{calcite}$ and $F_{aragonite}$). The calcite peak is larger than the corresponding aragonite peak (table S1), which is described using a proportionality constant $\beta_{calcite}$ which is experimentally determined in this calibration to be 4.97. Thus, the relationship between the relative amplitudes of the XRD peaks and the abundance of each mineral is given by the following equation.

$$A_{calcite} / (A_{calcite} + A_{aragonite}) = F_{calcite} * \beta_{calcite} / (F_{aragonite} + F_{calcite} * \beta_{calcite})$$

The results from this calibration study are displayed in Figure S1. The samples described in the main text (shown in Table S1) of this study differ by at most 1% when using this calibration and that of Swart et al., (2003), which is less than the generally accepted precision of this method (~5%).

Table S1. XRD peak areas for calcite [104] (A_{calc}) and aragonite [111] (A_{arag}) of calibration materials and bivalves from the CRNWR excavation site. Estimated calcite content for CNWR samples using this calibration and those estimated using the calibration of Swart et al., (2004) are provided

.

Reference Carbonate Materials (known mineralogy)

Sample ID	F_{arag}	F_{calc}	A_{arag}	A_{calc}	A_{calc} / [A_{arag} + A_{calc}]
A100 C000	100.0%	0.0%	100.26	0	0.0%
A100 C000	100.0%	0.0%	76.51	0	0.0%
A100 C000	100.0%	0.0%	89.7	0	0.0%
A075 C025	74.1%	25.9%	89.62	97.82	52.2%
A075 C025	74.1%	25.9%	70.12	143.45	67.2%
A075 C025	74.1%	25.9%	75.84	189.4	71.4%
A050 C050	46.8%	53.2%	48.47	276.16	85.1%
A050 C050	46.8%	53.2%	18.74	130.64	87.5%
A050 C050	46.8%	53.2%	56.21	285.31	83.5%
A025 C075	24.5%	75.5%	30.26	307.2	91.0%
A025 C075	24.5%	75.5%	30.56	295.54	90.6%
A025 C075	24.5%	75.5%	14.46	349.98	96.0%
A000 C100	0.0%	100.0%	0	505.07	100.0%
A000 C100	0.0%	100.0%	0	850.23	100.0%
A000 C100	0.0%	100.0%	0	299.83	100.0%

Bivalve samples from CRNWR_P13 Stratum I (unknown mineralogy)

Sample ID	A_{arag}	A_{calc}	A_{calc} / [A_{arag} + A_{calc}]
Level 1.1 A	90.5	5.55	5.8%
Level 1.1 B	113.51	0	0.0%
Level 1.1 C	45.58	0	0.0%
Level 1.1 D	152.55	0	0.0%
Level 1.1 E	101.09	0	0.0%
Level 1.1 F	99.32	0	0.0%
Level 1.2 A	119.61	0	0.0%
Level 1.2 B	112.58	0	0.0%
Level 1.2 C	81.62	10.33	11.2%
Level 1.2 D	34.09	0	0.0%
Level 1.2 E	91.23	1.93	2.1%
Level 1.2 F	54.49	0	0.0%
Level 1.3 A	105.18	0	0.0%
Level 1.3 B	27.21	0	0.0%
Level 1.3 C	92.62	0	0.0%
Level 1.3 D	246.34	0	0.0%
Level 1.3 E	121.77	0	0.0%
Level 1.3 F	62.55	0	0.0%
Level 1.4 A	108.54	0	0.0%
Level 1.4 B	57.14	9.06	13.7%
Level 1.4 C	60.43	19.6	24.5%
Level 1.4 D	72.7	6.54	8.3%
Level 1.4 E	84.28	11.39	11.9%
Level 1.4 F	103.34	7.04	6.4%

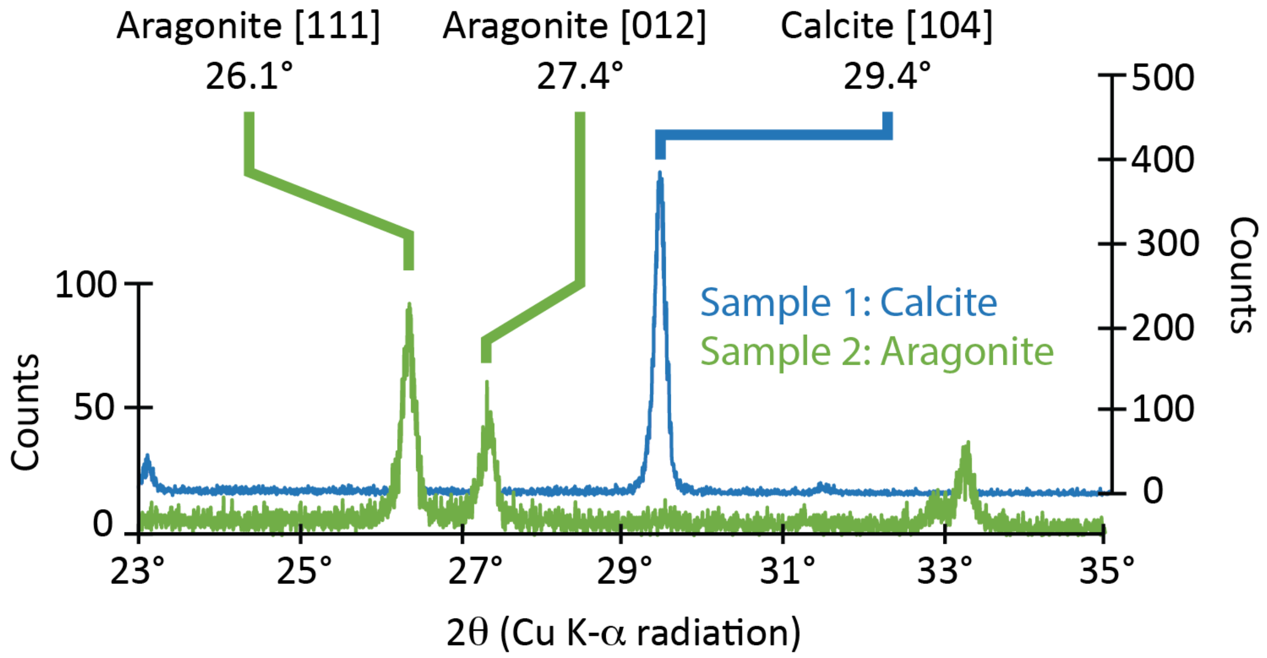

Fig. S1. XRD scans of pure aragonite and calcite. The aragonite [111] and calcite [104] peaks are used for quantification of mineralogy. Minor aragonite [121] and [012] peaks are visible at 2θ = 32.8° and 33.3° respectively.

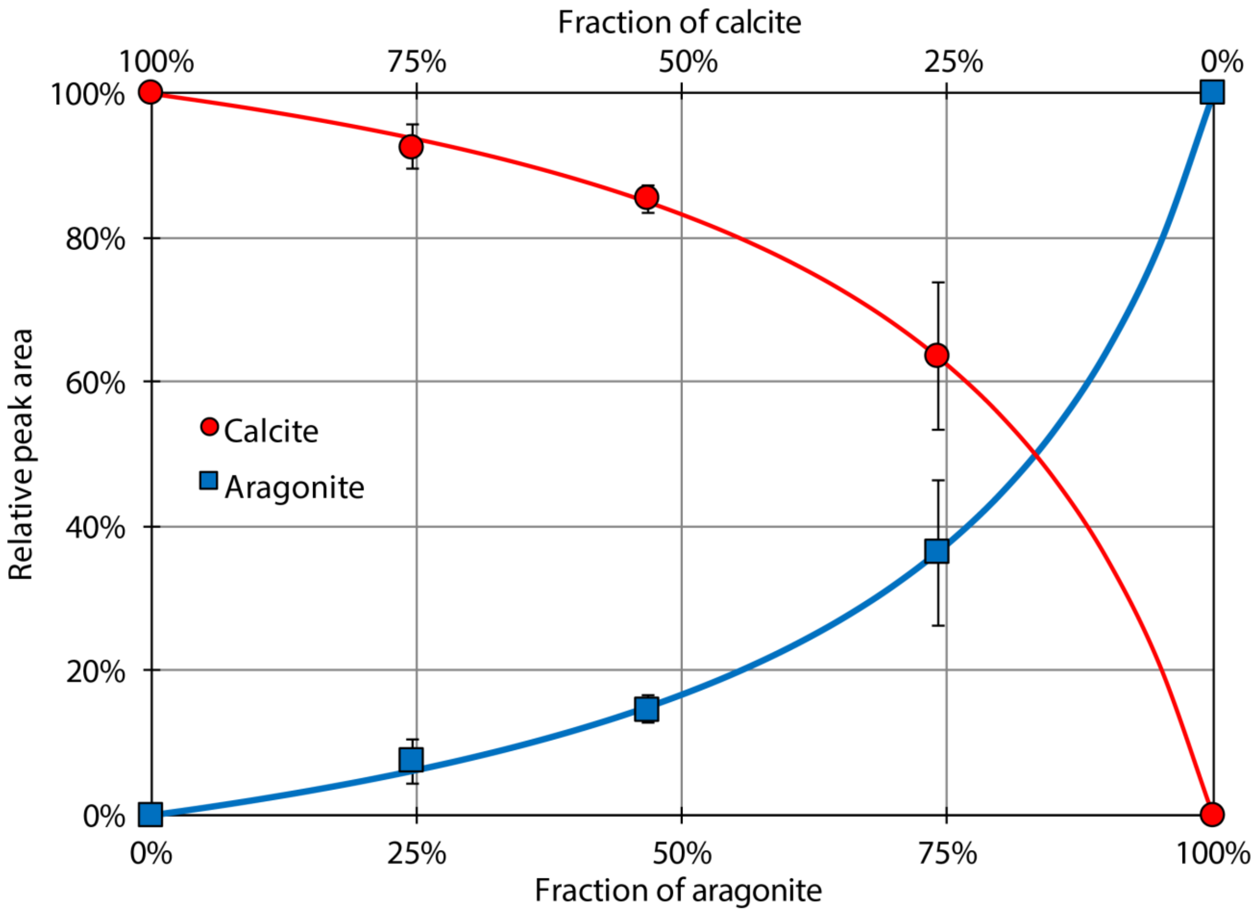

Fig. S2. Relative peak area for aragonite and calcite relative to the molar fraction of each mineral. Individual points and associated uncertainties describe the mean ± 1 standard deviation for three analyses. Solid lines show best fit model for available data.

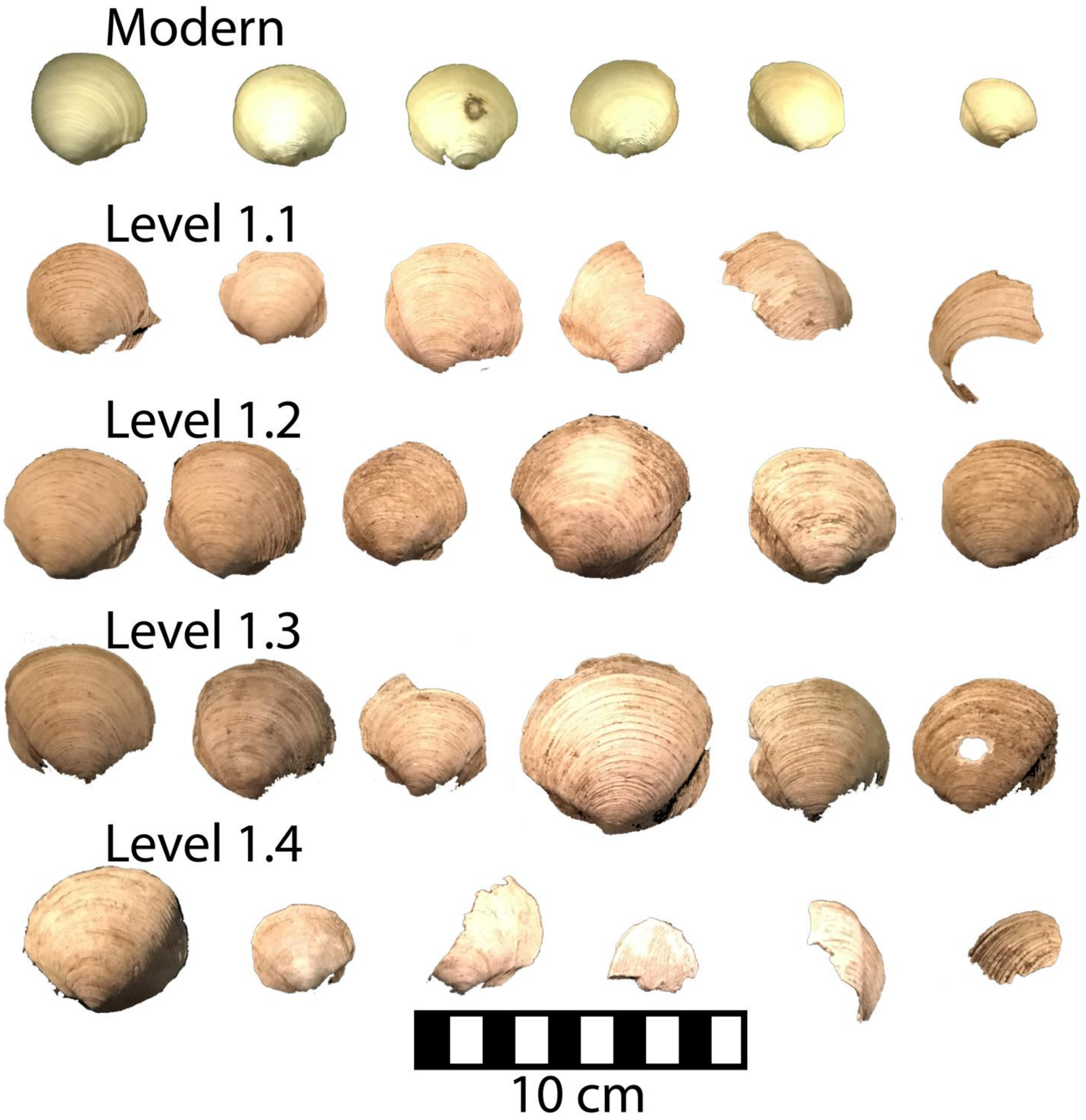

Fig. S3. Photographs of all shells analyzed in this study. Photo credit: Philip Staudigel, Cardiff University. Samples A-F are arranged from left to right for each level.

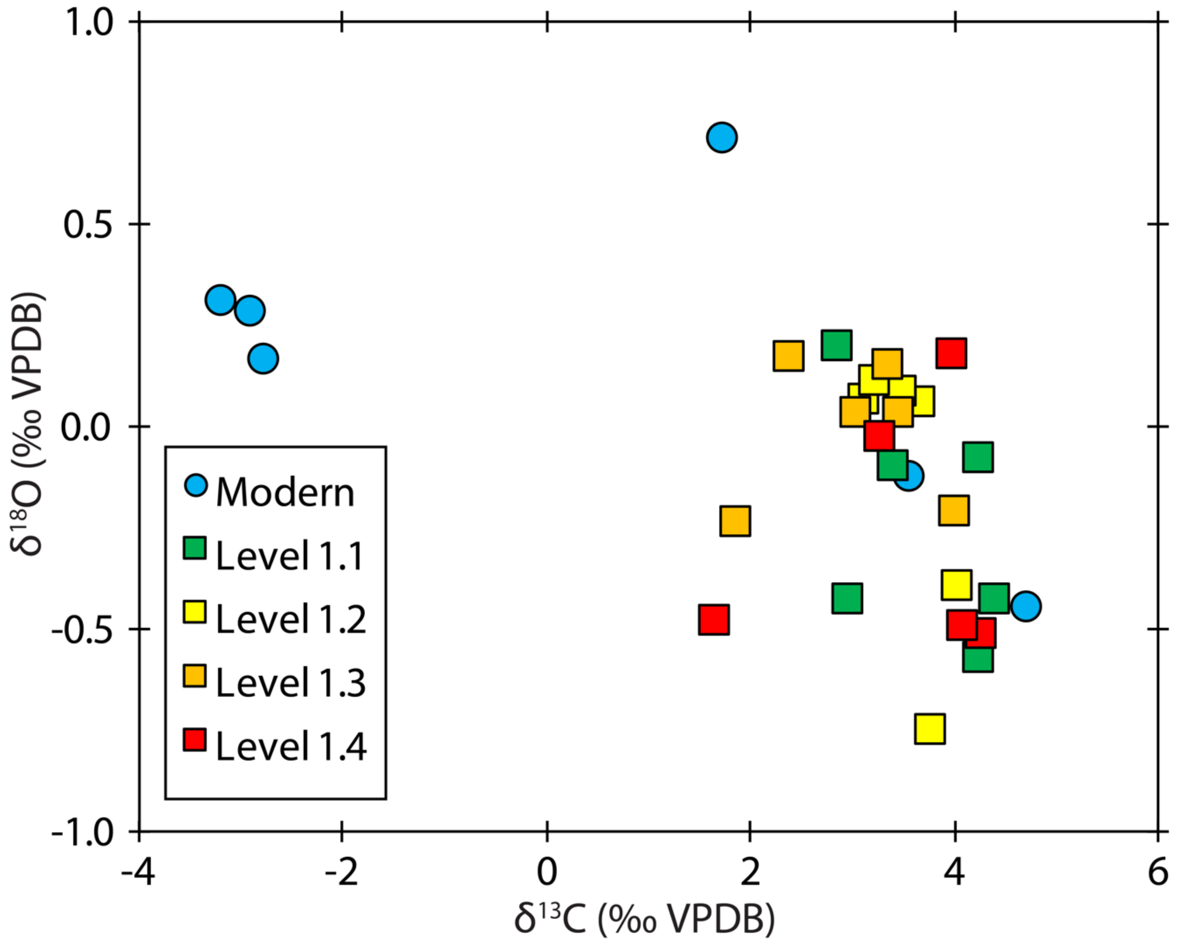

Fig. S4. Cross plot of $\delta^{13}C$ and $\delta^{18}O$ values for all modern and shell midden bivalves discussed in the main text.

Data S1. Isotopic analyses of equilibrated gases, Cabo Rojo bivalves, and ETH carbonate standards.
